# EHMTI-0115. Migraine under 7 years: a clinical study

**DOI:** 10.1186/1129-2377-15-S1-B25

**Published:** 2014-09-18

**Authors:** R Pitino, V Raieli, F Consolo, G La Franca, D Puma, G Santangelo, F Vanadia

**Affiliations:** 1 Child Neuropsychiatry School, University of Palermo, Palermo, Italy; 2 Child Neuropsychiatry Unit- Di Cristina Hospital, ARNAS CIVICO Palermo, Palermo, Italy

## Introduction

Migraine in children under 7 years has received limited attention and the few studies rarely report a careful description of clinical and therapeutic features.

## Aims

The aim of this study was to describe some characteristics of migraine phenotype in children under 7 years and to compare it with a population of migrainous children over 7 years.

## Methods

We reviewed all standard clinical files of children seen in a four years period and diagnosed with primary headache. In the study we included all children under 7 years age diagnosed with migraine. Twenty clinical variables were reported.

## Results

456 children with primary headaches (216 males, 240 females, mean age10.9 ± 3.1), have been seen during the study period. 374 children (188 males, 186 females) were affected by migraine with/without aura. The children under 7 years affected by migraine were 40 (8.9%) (20 males, 20 females, 5.7±1.2 mean age), two children had migraine with aura. These children showed generally a prevalence of main migrainous features similar to the group over 7 years. However the parameters found to be statistically significant different were duration of attacks and frequency of attacks (longer and more frequent with increasing age).

## Conclusion

our study shows that prevalence of migrainous characteristics is similar in younger to that of older migrainous, but duration of attacks and frequency of attacks are statistically different between two groups. These differences may reflect greater exhaustion of the mechanisms activating attacks with following refractoriness to a new attack.

No conflict of interest.

